# Immunohistochemical Detection of Receptor-Associated Protein in Normal Human Brain and Alzheimer's Disease

**DOI:** 10.4061/2010/173496

**Published:** 2009-12-20

**Authors:** John Provias, Brian Jeynes

**Affiliations:** ^1^Department of Pathology and Molecular Medicine [Neuropathology], Hamilton Health Sciences, McMaster University, Hamilton, ON, Canada L8L 2X2; ^2^Department of Community Health Sciences, Faculty of Applied Health Sciences, Brock University, St. Catharine's, ON, Canada L2S 3A1

## Abstract

This study is one of the few to characterize immunohistochemically the distribution and localization of Receptor-Associated Protein (RAP) in human autopsy brain. The results show prominent cortical neuronal localization. RAP is clearly identified in large neuronal dendritic/axonal processes. RAP is expressed in both large pyramidal and smaller interneurons. Occasional, much less frequent RAP is detectable in glial cells in white matter, which appear to be predominantly astrocytic. Although RAP is detectable immunohistochemically in Alzheimer's disease autopsy brain, the level of expression appears significantly reduced relative to age-matched control brains. These results suggest, at the immunohistochemical level, that there is a reduction of RAP protein in Alzheimer's disease brain (cortex). In terms of Alzheimer's disease pathophysiology, a reduction of neuronal RAP could then lead to reduced membrane expression of LRP, since RAP has also been shown to be an LRP antagonist.

## 1. Introduction

Receptor-associated protein (RAP) is a 39-kilo Dalton protein which is part of the large family of small GTPase proteins. It has also been shown to be a molecular chaperone for a number of receptor proteins, including low density lipoprotein receptor-related protein (LRP) [1–4]. LRP is a membrane protein which binds to amyloid precursor protein (APP), allowing its uptake and cellular internalization and subsequent processing to beta amyloid peptides [5, 6]. Alzheimer's disease is characterized by the excessive accumulations of beta amyloid peptide within brain regions, including areas of cortex. Beta amyloid is a peptide fragment derived from the larger precursor protein APP. The pathophysiology underlying the vast majority of Alzheimer's disease, that is, the sporadic nonfamilial cases, remains unknown. Given that RAP can modulate LRP function; it potentially could play a role in the transmembrane intracellular handling of APP and subsequent conversion to beta amyloid peptide. This clearly could be significant in terms of contributing to AD pathogenesis by affecting the beta amyloid peptide burden. RAP can also modulate the ligand binding activity of LRP through antagonism. As part of our ongoing studies examining mechanisms of beta amyloid clearance from human brain and our previous studies having examined LRP, as well as receptor for advanced glycosalation end (RAGE) products alterations in Alzheimer's brain, we decided to examine the immunohistochemical expression of RAP in both normal human and Alzheimer's cortex. There are only a few existing studies examining RAP in human brain of any type. One study has shown RAP to be present in cerebellar Purkinje cells [7]. Another study has observed a reduction of RAP in Alzheimer's relative to normative brains using biochemical means [8–10]. There are a number of lines of evidence suggesting a reduction of beta amyloid peptide clearance contributing to the accumulations which occur in Alzheimer's disease [11]. Therefore, any factor which alters or modulates beta amyloid uptake and neuronal processing, or microvascular clearance through modulations of other receptor proteins such as LRP, could be of significance. This study reports on our findings with immunohistochemical patterns of expression in Alzheimer's and a normative comparison group of normal brain cortices.

## 2. Materials and Methods

This study examined a cohort of 20 brains derived from the neuropathology autopsy service of Hamilton Health Sciences, McMaster University. Ten brains represented cases of Alzheimer's disease (AD), all with clinical dementia symptomology and neuropathologic confirmed typical Alzheimer's pathology, without significant confounding variables or other pathologic processes. In addition, there was a cohort of ten normative control brains (NM) free of significant cerebrovascular neuropathology or neurodegenerative process, without significant cognitive or dementia symptomology. All brains underwent a detailed neuropathologic examination by one of us (J.Provias, Neuropathologist), as well as CERAD [12] and Braak and Braak staging [13]. Standardized and comparable areas of superior temporal (ST) and occipital cortex (COC) were further examined from each brain and underwent immunohistochemical staining for receptor-associated protein (RAP). This was in addition to the standard neuropathologic workup utilizing immunohistochemistry for beta amyloid and tau. RAP immunohistochemistry was performed using a mouse monoclonal antibody at a working dilution of 1/200 (Bio Design International clone 7F1, Saco Me.). The distribution of RAP immunopositivity and the cellular characteristics were characterized in all cases and a semiquantitative assessment of the intensity of staining was determined using a 0–4+ scale. In each case we examined 10 contiguous ×400 magnification fields from the sampled cortices. The mean intensity values were determined based on the observations from the 10 fields per cortical sample. 

## 3. Results

**Figure 1:**
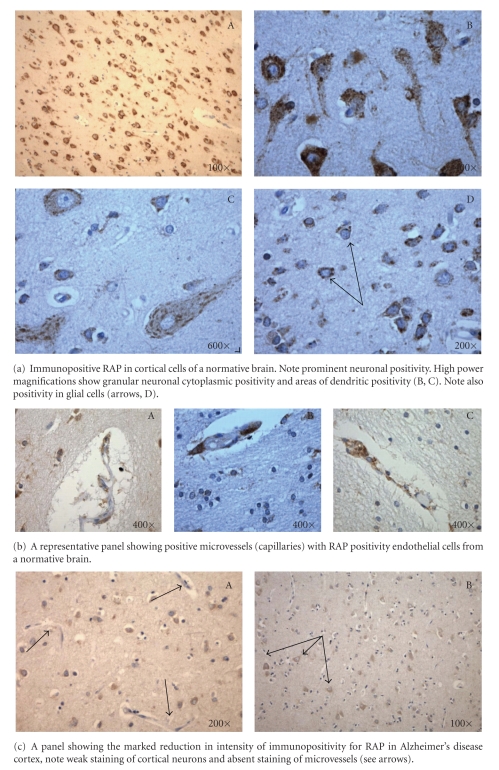
RAP immunohistochemistry in normal human cortex and Alzheimer's disease.

**Figure 2:**
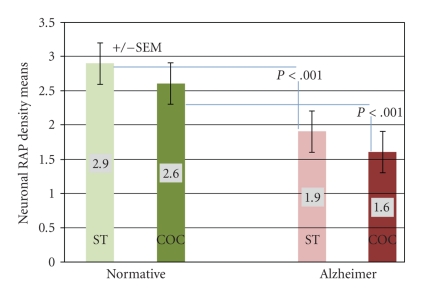
RAP immunmopositivity intensity-qualitative results. This bar graph summarizes the mean neuronal RAP density qualitative observations, and includes SEM values and *P*-values comparing ST and COC means between the Normative and AD conditions.

Table 1 summarizes the case descriptions. This study was meant to examine the expression of RAP in Alzheimer's disease cortex by looking at the temporal and occipital regions. It was not meant to look at expression in early versus late disease or in relation to disease progression. Therefore the selection of the AD cases includes brains with lower as well as higher Braak and Braak stages. Future studies employing a greater number of cases of each Braak and Braak stage will examine this latter point. Our results indicate that RAP can be detected immunohistochemically in autopsy human brain (Figure 1). There is strong cortical expression of RAP in a variety of cell types, both neurons and glia. Neurons show particularly intense immunopositivity with both larger pyramidal and smaller neurons being immunoreactive. The staining pattern was predominantly cytoplasmic, in some cases with a granular pattern present, consistent with endoplasmic reticular localization. Some neurons show prominent staining of neuritic processes. Subcortical white matter showed more restricted positivity with some focal positivity in glial cells. Further positivity was seen in some capillaries within endothelial cells. Within the Alzheimer's disease brains, the cortical positivity was present as in the normative, although less intense. Mean qualitative neuronal observation values, when observed on a scale of 0–4, was 2.9 and 2.6 in the superior temporal and calcarine cortices, respectively, in normative brains, and 1.9 and 1.5 in the superior temporal and calcarine cortices, respectively, in Alzheimer brains (Figure 2). No significant differences were observed between ST and COC values within each condition. However, significant differences (*P* < .001) were observed when comparing NM and AD conditions within comparable areas of the ST and COC areas. Of note, the pattern through Alzheimer's cortex was a diffuse cellular neuronal pattern without localization to senile plaques. The results indicate a reduced intensity of neuronal staining through the Alzheimer's disease cortex, both temporal and occipital, although with a similar qualitative cellular pattern of positivity. There was also a suggestion of reduced intensity of capillary vascular RAP positivity within the AD brains. Significant changes in glial RAP staining were not observed.

## 4. Discussion

This study is one of the very few which has examined RAP expression in human cortex. This study has demonstrated the immunohistochemical detection of RAP in both normal and Alzheimer's disease human cortex. There is greater expression in cortex than white matter, with the vast majority of cortical neurons showing strong immunopositivity irrespective of their neuronal size or morphology. RAP does not appear limited to neurons however, with detection being present in glial cells, particularly in subcortical white matter, some of which are clearly oligodendroglial cells. RAP is a 39-kilo Dalton protein which is thought to function as a molecular chaperone for other receptor proteins, in particular the LDL receptor protein family [1–4, 14]. The presence of RAP in cortical neurons would be consistent with its function as a modulator of low density lipoprotein receptor-related protein expression through its molecular chaperone functions. LRP is important as a major membrane receptor, allowing the binding and uptake of amyloid precursor protein, which in turn undergoes proteolytic cleavage to beta amyloid fragments [5, 6]. These beta amyloid fragments are one of the key pathologic abnormalities of Alzheimer's disease brain with excessive accumulation underlying the senile plaque pathology and thought by many to be a key initial causative event in the subsequent pathophysiologic chain of alterations occurring in Alzheimer's disease brain [5, 6]. Therefore, neuronal RAP by modulating LRP function and the uptake and processing of APP into beta amyloid could play a role in the overall modulation of the beta amyloid peptide burden of normal and Alzheimer's disease brain [10, 15–17]. However the distribution of RAP indicates a complexity as not only neuronal but also vascular and glial compartments contain RAP and could contribute to pathophysiologic alterations in Alzheimer's. The most striking change observed in these 10 cases was however the reduction in neuronal RAP with a suggestion of lesser reduction in vascular RAP. Future studies will have to look at these latter 2 compartments in more detail particularly as to how they may interact with neuronal RAP.

Though a preliminary qualitative study, this study suggests that in Alzheimer's disease cortex there is a reduction of RAP as indicated by reduced immunostaining. This change was seen in both temporal and occipital regions suggesting it may be widespread. This quantitative downregulation of RAP protein, in turn, could modify the LRP/APP/beta amyloid processing. A reduction of RAP in AD brain could reduce the LRP stability and membrane localization by virtue of its loss of chaperone function; this could be a protective or adaptive response in an attempt to reduce APP to beta amyloid processing. There is some evidence that RAP can also antagonize the LRP receptor [1–4, 10]; this would suggest that reduced neuronal RAP could lead to increased LRP receptor activity and APP internalization and possibly subsequent processing to beta amyloid peptide. There was a suggestion of some reduction of intensity of capillary endothelial RAP immunopositivity within the AD brains. However this was not as marked as the neuronal alterations seen. 

These results should be correlated in the future with biochemical analysis to confirm RAP reductions within Alzheimer's brain. They point towards the significance of alterations of modulating factors of the APP/beta amyloid processing pathway, potentially contributing to AD pathogenesis. 

## Figures and Tables

**Table 1:** Case descriptions.

Condition	Case	Gender	Age	Braak and Braak stage	Cerad level
Nondemented comparison cases	1	F	58	0	N/A
	2	F	72	0	N/A
	3	F	57	0	N/A
	4	M	75	0	N/A
	5	M	69	0	N/A
	6	M	45	0	N/A
	7	F	43	0	N/A
	8	M	41	0	N/A
	9	M	71	0	N/A
	10	F	74	0	N/A

Alzheimer cases	1	M	88	6	High
	2	F	75	6	High
	3	M	60	5/6	High
	4	F	74	6	High
	5	F	83	5/6	High
	6	M	74	3/4	High
	7	F	84	5/6	High
	8	M	84	5/6	Mod
	9	M	83	1/2	High
	10	M	74	3/4	High
